# Reference values of brachial-ankle pulse wave velocity according to age and blood pressure in a central Asia population

**DOI:** 10.1371/journal.pone.0171737

**Published:** 2017-04-12

**Authors:** Gulinuer Yiming, Xianhui Zhou, Wenkui Lv, Yi Peng, Wenhui Zhang, Xinchun Cheng, Yaodong Li, Qiang Xing, Jianghua Zhang, Qina Zhou, Ling Zhang, Yanmei Lu, Hongli Wang, Baopeng Tang

**Affiliations:** 1 Pacing and Electrophysiological Department, The First Affiliated Hospital of Xinjiang Medical University, Urumqi, Xinjiang, China; 2 Department of Cardiac Function, The First Affiliated Hospital of Xinjiang Medical University, Urumqi, Xinjiang, China; University of Colorado Denver School of Medicine, UNITED STATES

## Abstract

**Background:**

Brachial-ankle pulse wave velocity (baPWV), a direct measure of aortic stiffness, has increasingly become an important assessment for cardiovascular risk. The present study established the reference and normal values of baPWV in a Central Asia population in Xinjiang, China.

**Methods:**

We recruited participants from a central Asia population in Xinjiang, China. We performed multiple regression analysis to investigate the determinants of baPWV. The median and 10th-90th percentiles were calculated to establish the reference and normal values based on these categories.

**Results:**

In total, 5,757 Han participants aged 15–88 years were included in the present study. Spearman correlation analysis showed that age (r = 0.587, *p* < 0.001) and mean blood pressure (MBP, r = 0.599, *p* <0.001) were the major factors influencing the values of baPWV in the reference population. Furthermore, in the multiple linear regression analysis, the standardized regression coefficients of age (0.445) and MBP (0.460) were much higher than those of body mass index, triglyceride, and glycemia (-0.054, 0.035, and 0.033, respectively). In the covariance analysis, after adjustment for age and MBP, only diabetes was the significant independent determinant of baPWV (*p* = 0.009). Thus, participants with diabetes were excluded from the reference value population. The reference values ranged from 14.3 to 25.2 m/s, and the normal values ranged from 13.9 to 21.2 m/s.

**Conclusions:**

This is the first study that has established the reference and normal values for baPWV according to age and blood pressure in a Central Asia population.

## Introduction

In recent years, great emphasis has been placed on the role of aortic stiffness in the development of cardiovascular diseases. Increased arterial stiffness, particularly of the central arterial segments, has been demonstrated to be associated with increased cardiovascular risk and an independent predictor of stroke [1–5] With aging, stiffening of the arterial system may lead to an increase in aortic systolic pressure and subsequent stress on smaller arterial vessels, a decrease in aortic diastolic pressure, and left ventricular hypertrophy, consequently damaging the heart[6–10].

Pulse wave velocity (PWV) is known as a key indicator of arterial stiffness[10–11]. Although, PWV of the aorta has been used as the standard method of measuring arterial stiffness,PWV can be measured in other arterial regions,such as heart- brachial(hb), heart-carotid(hd),femoral-ankle(fa) segment. In recent years, especially in Japan and other Asian countries, brachial-ankle pulse wave velocity (baPWV) is now widely used for its simple, convenient and automatic method of measuring PWV [12–15]. Several studies have also shown that a high brachial-ankle pulse wave velocity (baPWV) is a predictor of mortality and cardiovascular events in different patient cohorts[16–22]. To verify whether baPWV is a surrogate end point for cardiovascular disease, Vlachopoulos et al. [17]performed a meta-analysis that involved 18 studies, and their results showed that an increase in baPWV by 1 m/s corresponded with an increase of 12%, 13%, and 6% in total cardiovascular events, cardiovascular mortality, and all-cause mortality, respectively.

Despite the great importance of baPWV in the clinical setting, a complete database of reference values is not yet available for the Central Asia population. The aim of this study was to investigate the major influencing factors of baPWV and to establish the reference values of baPWV that take these influencing factors into consideration in Xinjiang, China.

## Methods

### Ethical approval of the study protocol

This study was approved by the Ethics Committee of the First Affiliated Hospital of Xinjiang Medical University and was conducted according to the standards of the Declaration of Helsinki. Written, informed consent was obtained from the participants. We obtain the written informed consent from the next of kin, caretakers, or guardians on behalf of the minors/children enrolled in our study.

### BaPWV measurement

**Fig 1:**
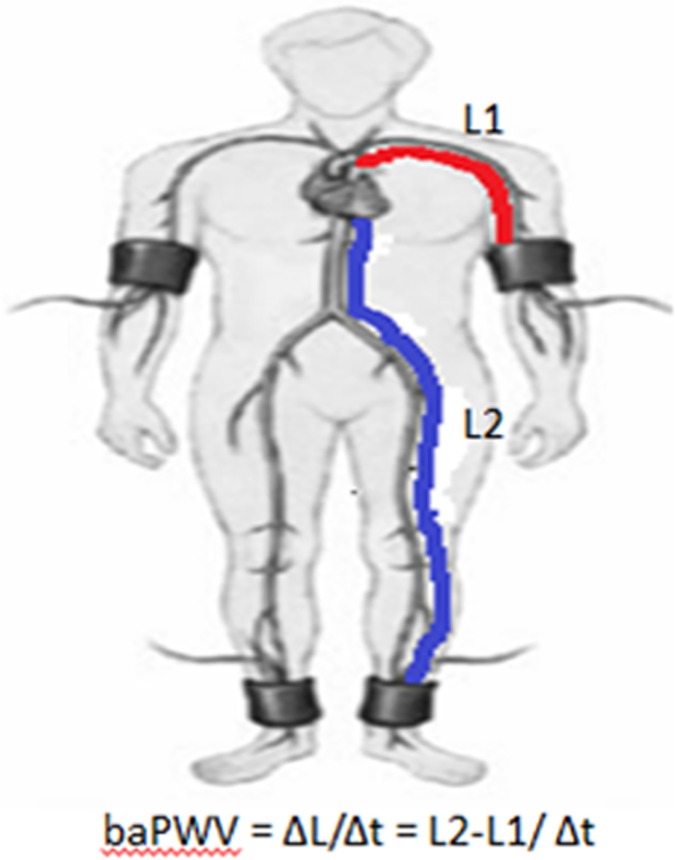
The working principle of baPWV measurement. The dictance from heart to the brachial arteries is marked by the red lines and is represented by L1. The dictance from heart to the ankle arteries is marked by the blue lines and is represented by L2. Thus, baPWV = ΔL/Δt = L2-L1/ Δt.

BaPWV was measured using the Vascular Profiler-1000 device (VP1000; Colin, Komaki, Japan) as previously described[23]. Briefly, VP1000 is a device with four cuffs that can measure blood pressure levels in the arms and legs and simultaneously record pulse waves using an oscillometric cuff technique. The device may estimate the travel path from body height and is able to compute baPWV automatically as the ratio of the travel path divided by the time difference between the pulse waves that are transmitted to the brachial and ankle arteries. This device is useful for mass medical examinations and studies because it enables measurements of baPWV in a short time without influence of the operator’s technique. The working principle is shown in Fig 1. Because of the significant positive correlation between the right and left baPWVs, we used the mean value of the right and left baPWVs during our analysis.

### Field work

Information on demographics, socioeconomic status, dietary habits, and medical history of each participant was collected by trained researchers using questionnaires. Height and body weight were measured as described previously. Three sitting blood pressure measurements were obtained using mercury sphygmomanometers after a 10-minute rest period, and the average of each measurement was used for data analysis. If the results differed by more than 5 mmHg, additional measurements were done. Mean blood pressure (MBP) was calculated from the systolic and diastolic blood pressures (SBP and DBP, respectively) as MBP = DBP + 0.4(SBP—DBP).

### Biochemical analyses

We measured the concentrations of serum total cholesterol (TC), triglyceride (TG), fasting glucose, low-density lipoprotein (LDL), high-density lipoprotein (HDL), using a biochemical analyzer (Dimension AR/AVL Clinical Chemistry System, Newark, NJ, USA).

### Definition of risk factors

Hypertension was defined as having an SBP ≥140 mmHg, DBP ≥90 mmHg, or if taking any antihypertensive medications. Furthermore, grade I hypertension was defined as an SBP ≥140 but ≤159 mmHg and a DBP ≥90 mmHg but ≤99 mmHg. Grade II hypertension was defined as an SBP ≥160 but ≤179 mmHg and a DBP ≥100 mmHg but ≤109 mmHg. Grade III was defined as an SBP ≥180 mmHg and a DBP ≥110 mmHg. Grade II/III hypertension was defined as an SBP ≥160 mmHg and a DBP ≥100 mmHg.

Participants were considered to have dyslipidemia if with TC concentration >5.2 mmol/L, TG concentration >1.7 mmol/L, LDL cholesterol concentration > 3.1 mmol/L, HDL cholesterol concentration <1.04 mmol/L, and/or taking a hypolipidemia drug.

Diabetes was defined as fasting plasma glucose level of at least 7.0 mmol/L, taking insulin or oral hypoglycemic agents, or a self-reported history of diabetes.

### Statistical analysis

Data analysis was performed using SPSS version 17.0 for Windows (SPSS, Chicago, IL, USA). Continuous variables were given as mean (standard deviation, SD), and the categorical variables were given as percentages. Correlations were assessed using multiple regression analyses. The influence of cardiovascular risk factors and sex on baPWV was examined by analysis of covariance, before and after adjustment for age and MBP. PWV values are represented as mean (SD) and median (10th-90th percentile). A high baPWV was defined as the 90th percentiles in each age and blood pressure category.

## Results

### Baseline characteristics of the study participants

**Fig 2:**
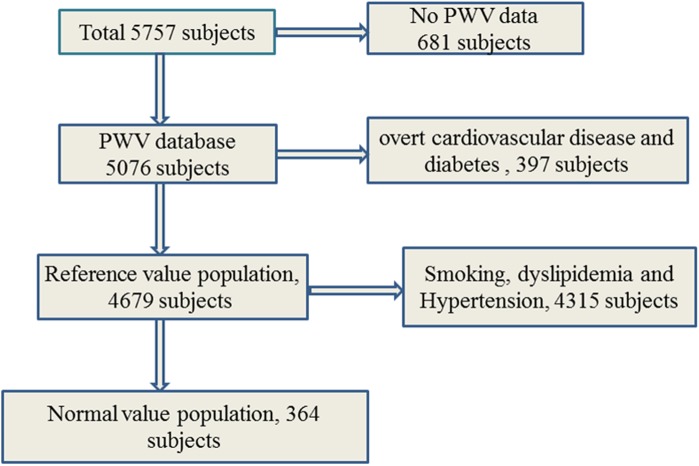
Flowchart describing the selection and categorization of subjects from the reference value database for the present analysis.

In total, 5,076 Han participants with complete data were enrolled in the present study, of whom 2,639 were male and 2,437 were female. The age of the participants ranged from 15 to 88 years, with a mean (SD) of 52.53 (12.65) years[24, 25]. Table 1 shows the demographic parameters and clinical data of the total population, reference value population, and normal value population. Total population was defined as Han participants with complete data. A total of 4,679 participants without overt cardiovascular disease and diabetes from the total population were assigned as the reference value population; 364 participants from the reference value population without dyslipidemia and are nonsmokers were assigned as the normal-value population. The detail methodology behind the recruitment is as Fig 2.

**Table 1:** Characteristics of study population.

Parameter	Total population	Reference value population	Normal value population
**Number**	5076	4679	364
**Age(years)**	52.53(12.65)	52.13(12.59)	44.95(9.66)
**Age range(years)**	(15–88)	(22–88)	(25–82)
**Male(%)**	2639(52)	2465(52.7)	300(82.4)
**BMI(kg/m2)**	25.11(3.51)	25.02(3.51)	23.06(2.79)
**SBP(mmHg)**	132.68(19.92)	131.75(19.56)	116.52(10.51)
**DBP(mmHg)**	85.12(15.58)	84.65(15.41)	74.52(9.48)
**MBP(mmHg)**	104.14(15.89)	103.49(15.66)	91.32(8.33)
**TG(mmol/L)**	1.70(1.40)	1.62(1.22)	0.90(0.33)
**TC(mmol/L)**	4.69(1.08)	4.66(1.07)	4.05(0.73)
**HDL-C(mmol/L)**	1.25(0.45)	1.25(0.45)	1.46(0.32)
**LDL-C(mmol/L)**	2.87(0.91)	2.88(0.91)	2.44(0.46)
**Glycaemia(mmol/L)**	5.32(1.78)	4.95(0.73)	4.73(0.69)
**Smoking(%)**	1605(31.6)	1457(31.1)	
**Hypertention(%)**	1985(39.1)	1748(37.4)	
**Dyslipidemia(%)**	4063(80.04)	3719(79.48)	
**Diabetes(%)**	397(7.8)		

y, years; SD, standard deviation; BMI, body mass index; SBP, systolic blood pressure; DBP, diastolic blood pressure; MBP, mean blood pressure; TG, triglyceride; TC, total cholesterol; HDL-C, high-density lipoprotein cholesterol; LDL-C, low-density lipoprotein cholesterol.

### Multiple linear regression analyses on baPWV

Before establishing the reference values for baPWV, the major determinants of baPWV were examined. Spearman correlation analyses were performed between baPWV and age, body mass index (BMI), MBP, TG, TC, HDL-C, LDL-C, and glycemia in the reference value population (Table 2). Multiple regression analysis using baPWV as a dependent variable was also performed in the reference value population (Table 3). The linear regression equation is as follows: baPWV (m/s) = -292.9 + 13.3 * age (years) - 5.8 * BMI (kg/m^2^) + 11.1 * MBP (mmHg) + 10.8 * TG (mmol/L) +16.7 * glycemia (mmol/L). This equation explains 53.2% of the baPWV variance.

**Table 2:** Spearman correlation analysis on brachial-ankle pulse wave velocity (baPWV).

Variable	r value	P value
**age**	0.587	<0.001
**BMI**	0.197	<0.001
**MBP**	0.599	<0.001
**TG**	0.104	<0.001
**TC**	0.107	<0.001
**HDL-C**	-0.011	0.442
**LDL-C**	-0.03	0.037
**Glycaemia**	0.091	<0.001

baPWV, brachial-ankle pulse wave velocity; BMI, body mass index; MBP, mean blood pressure; TG, triglyceride; TC, total cholesterol; HDL-C, high-density lipoprotein cholesterol; LDL-C, low-density lipoprotein cholesterol.

**Table 3:** Multiple regression analysis using baPWV as dependent variable.

Variable	Non-standardiz	standardiz	t	p value	R2
**Constant**	-292.898		-7.466	<0.001	0.532
**Age**	13.334	0.445	41.627	<0.001	
**BMI**	-5.831	-0.054	-4.956	<0.001	
**MBP**	11.092	0.46	40.679	<0.001	
**TG**	10.831	0.035	3.315	0.001	
**Glycaemia**	16.718	0.033	3.139	0.001	

baPWV, brachial-ankle pulse wave velocity; BMI, body mass index; MBP, mean blood pressure; TG, triglyceride.

Table 4 shows the analysis of covariance on baPWV. Before adjustment, the univariate analysis showed that baPWV was significantly higher in males, those with dyslipidemia and/or diabetes, and smokers (all *p* <0.001). However, after adjustment for age and MBP, there was no significant effect of sex, dyslipidemia, and smoking on baPWV (*p* = 0.316, 0.652, and 0.215, respectively). Only diabetes was a significant independent determinant of baPWV (*p* = 0.009). Therefore, participants with diabetes were excluded from the reference value population.

**Table 4:** Inflence of gender, dyslipidemia, Diabetes and smoking on baPWV(m/s) before and after adjustment for age and mean blood pressure (MBP).

	subjects	Mean age	P	MBP	P	PWV before adjustment		PWV after adjustment	
						Mean	P value	Mean	P value
Gender									
Female	103	51.80(14.33)	<0.001	102.61(12.36)	<0.001	14.9	<0.001	13.7	0.316
Male	364	46.01(9.83)		95.02(12.79)		13.2		13.5	
Dyslipidemia									
NO	467	47.28(11.22)	<0.001	96.70(13.07)	<0.001	13.5	<0.001	14.8	0.652
YES	2510	52.94(12.35)		103.34(15.94)		15.1		14.9	
Diabetes									
NO	467	47.28(11.22)	<0.001	111.56(14.76)	<0.001	13.5	<0.001	13.8	0.009
YES	39	57.33(12.04)		127.79(19.32)		17.3		14.7	
Smoking									
NO	467	47.28(11.22)	<0.001	96.7(13.07)	<0.001	13.5	<0.001	14.1	0.215
YES	248	53.95(14.35)		103.02(15.73)		15.3		14.3	

baPWV, brachial-ankle pulse wave velocity; MBP, mean blood pressure; PWV, pulse wave velocity.

### Reference and normal values for PWV

Table 5 shows the mean and reference values of baPWV according to age and MBP in the reference value population. Both mean values and reference values were observed to increase together with increasing age and blood pressure categories. The mean values ranged from 12.3 to 20.3 m/s, and the reference values ranged from 14.3 to 25.2 m/s. Table 6 shows the mean and normal values of baPWV in the normal value population. The mean values range from 12.0 to 16.3 m/s, and the normal values, from 13.9 to 21.2 m/s.

**Table 5:** Reference value of baPWV(m/s) according to age and blood pressure.

Age class(years)	Blood pressure class		
	Normal	GradeⅠ	GradeⅡ/Ⅲ
**baPWV as mean(±2SD)**			
**<40**	12.3(9.3–15.3)	14.3(10.7–17.9)	15.4(10.8–20)
**40–49**	12.7(10.1–15.3)	14.1(6.1–22.1)	15.7(10.5–20.9)
**50–59**	13.9(10.1–17.7)	16.1(10.9–21.3)	17.1(10.7–23.5)
**>60**	16.0(10.6–21.4)	18.4(11.8–25.0)	20.3(11.7–28.9)
**baPWV as Median(10-90th)**			
**<40**	12.2(10.4–14.3)	14.1(11.9–16.3)	15.0(13.2–19.8)
**40–49**	12.5(10.8–14.6)	13.8(11.7–16.6)	15.2(12.8–19.0)
**50–59**	13.5(11.8–16.4)	16.0(12.9–19.2)	16.4(13.8–21.6)
**>60**	15.7(12.8–19.2)	17.8(14.8–22.4)	19.6(15.9–25.2)

baPWV, brachial-ankle pulse wave velocity; SD, standard deviation.

**Table 6:** Normal values of baPWV(m/s).

Age class(years)	n	Mean(±2SD)	Median(10-90th)
**<40**	94	12.0(9–15)	11.7(10.3–13.9)
**40–49**	185	12.0(9–15)	11.9(10.4–14.0)
**50–59**	49	13.9(10.1–17.7)	13.6(12.1–16.4)
**>60**	36	16.3(10.3–22.3)	16.4(13.1–21.2)

baPWV, brachial-ankle pulse wave velocity; SD, standard deviation.

## Discussion

Lesions in arterial vascular structures marked by atherosclerosis and arteriosclerosis are the basis of various cardiovascular diseases[4, 9]. As changes in arterial stiffness occur earlier than those in arterial vascular structures, detection of arterial stiffness may play an important role in the prevention of a series of major cardiovascular diseases[3]. Several studies have demonstrated that PWV is an independent predictor of coronary heart disease and stroke in seemingly healthy subjects [16, 26].baPWV is a new and noninvasive measurement of arterial stiffness and has been widely used in health examinations and screenings in East Asian countries [14, 15]. baPWV has been demonstrated to be associated with population, age, blood pressure, and many other risk factors. The present study determined and evaluated for the first time the reference values of baPWV according to age and blood pressure in Xinjiang, China. In addition, the association between baPWV and cardiovascular disease risk factors was investigated.

Previous studies have demonstrated that traditional atherosclerotic risk factors such as diabetes mellitus, dyslipidemia, smoking, and hypertension may influence baPWV [27, 28]. An increasing number of studies have recently reported that baPWV increases with age, and this is mainly due to degeneration and decreased elasticity of the arterial wall [29]. Miyai et al. [30]claimed that age was one of the significant determinants of baPWV in a Japanese population, and Ai et al. [21]had the same conclusion in a Chinese population. Meanwhile, a study including 131 apparently healthy Japanese without a history of cardiovascular disease suggested that both age (*p* = 0.022) and blood pressure (*p* < 0.001) were the strongest independent determinants of baPWV [31]. The Framingham study performed by Mitchel G et al suggested that higher pulse pressure at any age and higher pulse pressure with advancing age is predominantly associated with PWV [13]. In the present study, baPWV was relatively strongly associated with age and blood pressure, but a weak association exists between baPWV and BMI, TG, and glycemia according to the multiple regression analysis. This indicates that age and blood pressure play a key role in baPWV. Hence, we considered age and MBP when establishing the reference values of baPWV in the present study.

The cutoff points of high baPWV can be represented in different ways. Important differences exist among ethnicities, and many cardiovascular risk factors may influence baPWV [22]. As mentioned above, baPWV is greatly influenced by age and blood pressure; thus, we should take these two confounders into consideration when establishing cutoff points of high baPWV. Otherwise, the prevalence of “high baPWV” would increase dramatically with increase in age and blood pressure. Thus, the cutoff points of high baPWV are represented by age and blood pressure categories.

Furthermore, we performed covariance analysis to verify whether other cardiovascular risk factors also influence baPWV after adjustment for age and blood pressure. The result of the univariate analysis showed that significant difference exists in baPWV between males and females, between those with dyslipidemia and normal lipid levels, and between smokers and nonsmokers. However, after adjustment for age and MBP, the difference disappeared, and this may be due to the different ages and MBPs in each subgroup. The study by Adams MR et al suggested that the increasing of estrogen will inhibit the formation of atherosclerotic plaques and reduce arterial stiffness [32]. Rajkumar C et al peformed a study and found that long-term hormonal therapy in postmenopausal woman significantly reduces aorto-femoral PWV and increases systemic arterial compliance compared with age-match control subjects not using such therapy [33]. Thus some researchers suggested that sex may influence the PWV. While, others had the different opinion. Both Smulyan H and Mattaee peformed the relevant researches and suggested that there exists no significant difference of PWV between male and female [34]. In our study, after adjustment for age and MBP, the difference of baPWV between male and female disappeared. The reason for the different results may be that we should adjust for influencing factors of baPWV when analysising the relationship between sex and baPWV. However, baPWV was significantly higher in subjects with diabetes than in normal subjects after adjustment for age and MBP. All the data above indicated that sex, lipid status, and smoking could not influence baPWV independently after adjustment for age and MBP; however, diabetes was a significant independent determinant of baPWV. Hence, the reference value population did not include participants with diabetes.

In accordance with the consideration above, we established the reference values and normal values according to age and blood pressure. The values of baPWV were represented as means and medians simultaneously. High baPWV was defined as the 90th percentiles in each age and MBP category. In the previous studies, several fixed threshold values of baPWV had been suggested. Yamashina et al. [12]defined 1,400 cm/s as a cutoff point to discriminate patients with either stroke or coronary heart disease. Han et al. [19]demonstrated that the baPWV level (cutoff, 1,704 cm/s) was an independent predictor of cardiovascular events, especially ischemic stroke in the general population. However, once these threshold values were used in the clinical setting, a large number of elderly or hypertensive subjects were diagnosed as having high baPWV, which means that 47.9% (2,239/4,679) of the subjects of the present study are at risk. Obviously, it is more precise to establish reference values according to age and blood pressure than fixed thresholds.

baPWV has been increasingly used as an index of arterial stiffness in recent clinical research studies. The results of some studies suggested that baPWV can not only provide qualitatively similar information to those derived from aortic PWV but also arterial stiffness of peripheral arterial. While carotid-to-femoral/carotid-to-radial PWV can only reflect the aortic PWV [12]. This is the difference between baPWV and cfPWV/crPWV. However, although aortic PWV is accurate, it may not be ideal for routine use in cross-sectional study because of the repeatability and difficulty of measuring cfPWV. Additionally, some subjects may feel uncomfortable exposing the inguinal area during the acquisition of femoral pressure waveforms. On the contrary, baPWV is a relatively simpler method to measure PWV, It just requires patients placing blood pressure cuffs on the four extremities, and the Vascular Profiler-1000 device may calculate the baPWV automaticly [14]. The method using PC-MRI to measure PWV is a more accurate techniques. It is a intuitive and objective way with less estimation to measure PWV [15]. Though it has so many advantages, it exists some disadvantage too. First of all, the method using PC-MRI to measure PWV cost too much, most people can not afford the cost in China. Second, it has a more complex operation procedure than baPWV, and is not suitable for cross-sectional study. Additionally, people with MRI contraindication can not measure PWV in this way. Compared with cfPWV and PC-MRI-based mwthod, though baPWV has many shortages, it is still a more convenient and economical way to estimate arterial stiffness in cross-sectional study.

In conclusion, age and blood pressure are the most important factors influencing baPWV. The other factors associated with baPWV were BMI, TG, and glycemia. The reference and normal values of baPWV in the Central Asia population were established according to age and blood pressure in the present study. It is now possible to estimate whether subjects have a high baPWV in a certain age and blood pressure category.

## Supporting information

S1 Table Raw data. Complementary information on raw data.
(XLSX)
